# Deep Convolutional Neural Network for Nasopharyngeal Carcinoma Discrimination on MRI by Comparison of Hierarchical and Simple Layered Convolutional Neural Networks

**DOI:** 10.3390/diagnostics12102478

**Published:** 2022-10-13

**Authors:** Li Ji, Rongzhi Mao, Jian Wu, Cheng Ge, Feng Xiao, Xiaojun Xu, Liangxu Xie, Xiaofeng Gu

**Affiliations:** 1 Department of Otorhinolaryngology, The Second People’s Hospital of Changzhou Affiliated to Nanjing Medical University, Changzhou 213003, China; 2 Institute of Bioinformatics and Medical Engineering, School of Electrical and Information Engineering, Jiangsu University of Technology, Changzhou 213001, China

**Keywords:** nasopharyngeal carcinoma, deep learning, image diagnosis, convolutional neural network, artificial intelligence

## Abstract

Nasopharyngeal carcinoma (NPC) is one of the most common head and neck cancers. Early diagnosis plays a critical role in the treatment of NPC. To aid diagnosis, deep learning methods can provide interpretable clues for identifying NPC from magnetic resonance images (MRI). To identify the optimal models, we compared the discrimination performance of hierarchical and simple layered convolutional neural networks (CNN). Retrospectively, we collected the MRI images of patients and manually built the tailored NPC image dataset. We examined the performance of the representative CNN models including shallow CNN, ResNet50, ResNet101, and EfficientNet-B7. By fine-tuning, shallow CNN, ResNet50, ResNet101, and EfficientNet-B7 achieved the precision of 72.2%, 94.4%, 92.6%, and 88.4%, displaying the superiority of deep hierarchical neural networks. Among the examined models, ResNet50 with pre-trained weights demonstrated the best classification performance over other types of CNN with accuracy, precision, and an F1-score of 0.93, 0.94, and 0.93, respectively. The fine-tuned ResNet50 achieved the highest prediction performance and can be used as a potential tool for aiding the diagnosis of NPC tumors.

## 1. Introduction

Nasopharyngeal carcinoma (NPC) is the 23rd most common cancer in the world according to the 2018 Global Cancer Statistics [1]. NPC occurs more often in certain parts of South Asia, the Middle East, and North Africa. As one of the most common cancers, its diagnosis and therapy attract extensive attention [2]. Survival rates differ significantly between NPC patients in the early stages and late stages, which can be classified as stages T1 to T4. The ten-year survival rate of patients with early-stage (stage T1) is around 98%, whereas survival of stage T2 drops to 60% [3], implying the importance of early diagnosis for successful treatment. However, NPC usually has no specific symptoms at the early stage.

Many early diagnosis approaches are under development. For example, Epstein-Barr virus (EBV) and consumption of certain foods including salted fish and preserved foods are reported to be associated with high risks of NPC [4]. EBV serological biomarkers and fiberoptic endoscopy/biopsy enable early diagnosis of NPC. However, identifying the biomarker is labor-intensive. Other diagnosis methods of NPC include physical examination, nascopharygoscopy, and imaging. As one convenient approach, medical imaging examinations are widely used in diagnosis, such as computed tomography (CT), magnetic resonance imaging (MRI), or positron emission tomography (PET) [5]. Medical imaging examinations provide detailed information on NPC for specialists. However, the lesion of NPC presents variable shapes, sizes, and locations. The lesion may even occupy only a very small fraction of the whole image series. Medical image interpretation has been performed solely by radiologists in the clinic, which requires professional experience and, meanwhile, brings high labor and time costs.

Artificial intelligence (AI) has seen significant developments in academia and industry. The applications of AI have achieved great breakthroughs in many fields, such as cheminformatics, drug design, and AI-assisted medical imaging [6]. AI plays an important role in medical data analysis, medical diagnosis, and healthcare [7,8,9]. Particularly, successful applications have been reported in medical imaging, such as image classification, image segmentation, object detection, image generation, and image transformation [10,11,12,13,14,15]. It is still challenging to obtain high-quality open databases. To overcome this problem, image generation and image transformation methods have been developed [16,17]. As one popular application of AI-assisted diagnosis, image classification can automatically classify medical images into benign and malignant, or differentiate the stage of disease [18]. Moreover, AI greatly speeds up disease detection and the segmentation of the pneumonia lesions of COVID-19 [19]. According to a recent review of deep learning in medical imaging, applications have focused on several major fields: ophthalmology imaging, respiratory imaging, breast imaging, and other specialties [20]. Notwithstanding, the concern should be pointed out that previous studies have put more emphasis on the major disease, and attempts of implementing AI in particular minor specialties are still in their infancy.

Several computer-assisted disease diagnoses of NPC have been developed from machine learning approaches to deep learning approaches. Medical images of NPC, such as CT and MRI, can be processed by AI methods [21]. Researchers have adopted traditional machine learning (ML) based medical image analysis of NPC. The generally used ML methods include artificial neural network (ANN), k-nearest neighbor (KNN), random forest (RF), and support vector machine (SVM) models, etc. Lu et al. reported that ML methods can be used to differentiate local recurrence versus inflammation from post-treatment nasopharyngeal positron emission tomography/X-ray computed tomography (PET/CT) images [22]. Zhang et al. obtained the highest mean area under the curve (AUC) of 0.846 from the selected features of MRI by using RF [23]. However, feature selection is required in traditional machine learning methods. The purpose of feature selection is to reserve meaningful features extracted from original images. The appropriate feature selection is critical for the performance of machine learning models. Consequently, extra efforts need to be conducted before identifying the optimal combination of feature selection methods and ML methods. For example, Zhang et al. conducted systematic research on combinations of six feature selection methods and nine ML classification methods [23]. Recent studies show that there is still invisible information that remains to be discovered while traditional feature selection methods cannot capture all of the information, especially for high-dimensional information [24]. The existing works on NPC classification have adopted traditional machine learning methods, such as SVM, ANN, and simple CNN, etc. Acceptable accuracy has been achieved, however, feature selection is required. The detailed strengths and weaknesses of models can be found in the recent literature [25,26,27,28,29].

Deep learning (DL) has been reported to show the capability of feature selection and can train algorithms to automatically determine important features [30,31]. Instead of extracting features manually, DL can learn the informative representations in a self-taught manner. The unique advantages of automatic feature selection make deep learning one powerful tool for analyzing high-dimensional image data. DL started attracting increased interest when the convolutional neural network (CNN) surpassed human performance in the ImageNet Large-Scale Visual Recognition Challenge (ILSVRC) in 2012 [32]. CNN was originally designed to solve the computer vision problem that makes computers recognize objects from natural images. CNN can easily extend its applications in medical imaging. Yeh et al. used CNN to successfully identify NPC in nasopharyngeal biopsies [33]. Qian et al. reported a DL-based automatic diagnosis system for identifying NPC from noncancer using both white light imaging and narrow-band imaging nasopharyngoscopy images [34]. Xia et al. proposed NPC-Seg for the segmentation of NPC using computed tomography (CT) [35]. Sun et al. applied CNN to the automated contour of primary tumor volumes by MRI, indicating the success of AI-based tools in detecting NPC tumors [36]. Few studies have been conducted to explore the generalizability and reliability of the latest CNN on the classification task of MRI images of NPC. Meanwhile, DL models suffer from issues in choosing suitable architecture and searching for the best set of hyper-parameters during optimization [37]. Therefore, choosing a suitable network for a given problem is still a challenging task. 

Compared with other medical imaging, MRI is commonly adopted as a routinely used protocol for the diagnosis of NPC because of its non-invasiveness and its high efficiency. MRI is the most commonly used radiographic method for diagnosis besides the endoscopic image. The application of deep neural networks needs to be fine-tuned based on specific requirements. To verify the effectiveness of deep learning models for identifying NPC from non-NPC, this study investigated the performance of the latest established hierarchical CNN models. As proved, ResNet and EfficientNet gain great improvement in the classification task of ImageNet than the previous reported CNN-based models. It would be worthy of study to apply hierarchical CNN models to the specific classification of NPC in order to harness the feature learning ability of deep learning methods. To the best of our knowledge, hierarchical CNN models, such as ResNet and EfficientNet, have not been applied in NPC discrimination. In this study, we examined the performance of shallow and deep convolutional neural networks in order to identify one optimal model for automatic diagnosis from MRI images of NPC by comparing the accuracy, precision, and F1-score of each model. Due to the lack of a high-quality NPC image database, we collected MRI images of patients and manually built the tailored NPC image dataset. We first performed hyper-parameter optimization for each model. Then, we compared the performance of sCNN, ResNet50, ResNet101, and EfficientNet-B7 without and with the pre-trained weights. Finally, we applied the gradient-weighted class activation map (Grad-CAM) to visualize convolutional layers in order to provide interpretable information for classification.

## 2. Materials and Methods

### 2.1. Dataset Preparation

The original MRI images from different imaging planes and the proportion of effective images that can be used for the diagnosis are small. Generally, the critical factor in the successful application of deep learning is to construct a high-quality dataset. MRI images of NPC were collected from our hospital. MRI images in DICOM format were collected from 52 subjects. Fast-spin echo (FSE) axial T1-weighted images and contrast-enhanced FSE axial T1-weighted images were scanned at 1.5 T. The ages of the patients range from 27 to 74 years old. The collected data period lasted from 2018 to 2021. The section gaps range from 2.0–9.0  mm along the scanning planes.

**Figure 1:**
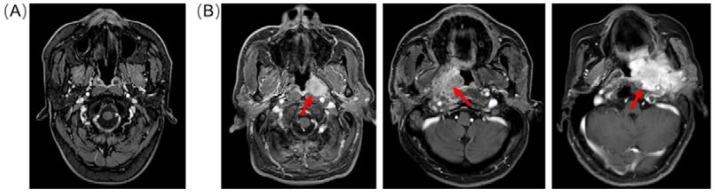
Example of contrast-enhanced T1-weighted MRIs. (**A**) The image from normal control without NPC cancer; (**B**) the images from patients with varying sizes of NPC. The arrows indicate the location of the NPC tumor.

Axial contrast-enhanced T1-weighted images are routinely used to detect tumor extension and provide more clinical value for NPC, such as perineural spread and intracranial extension of tumors. Therefore, axial contrast-enhanced T1-weighted images were chosen for this study. The personal information was removed from the original MRI images, such as the patient’s name, the patient’s age, and the series description, etc. Figure 1 shows the axial MRI images of the NPC patients. The fossa of Rosenmuller is the most common site of origin for NPC. We selected 9–39 slices around the original site of the nasopharynx space from the MRI images depending on the different slice thicknesses. In order to make the NPC image dataset compact, the images containing invaded organs were selected for the confirmed patients under the supervision of the radiologist. After screening the images, we collected 972 MRI images from 20 confirmed patients and 20 control by balancing the number of positive and negative samples. The images were further preprocessed by data augmentation with default settings implemented in Keras. The images were normalized to 0–255 and then the input shape was reshaped from 512×512×3 into 150×150×3 in order to save GPU memory by the ImageDataGenerator of Keras.

### 2.2. Choice of Splitter

For each patient, multiple image slices would be obtained from the MRI. The image-level contextual splitter has been used by previous classification algorithms. For a general random train-test splitter, the images from the patients were mixed. The images from an individual patient may be separated into a training and a test set by only using image-level contextual information. Though images were different in the image series of the MRI along the scanned plane, the neighboring images in the MRI image sequences shared a high similarity. However, the random splitter based on image-level contextual information may lead to information leakage during the training process. Hence, we decided to divide the dataset by including the patient-level contextual information. That is to say, images from one patient can only be split into one category (training set or test set) during each training process. The patient index and the image index were then used to divide the train-test dataset in the “model_selection” method in the Scikit-learn package when using a patient-level splitter. The images were then split into a train and test set with a ratio of 9:1. For comparison, the image- and patient-level contextual information was adopted for the splitter when training the shallow and deep neural networks.

### 2.3. Evaluated Shallow and Hierarchical Convolutional Neural Network

**Figure 2:**
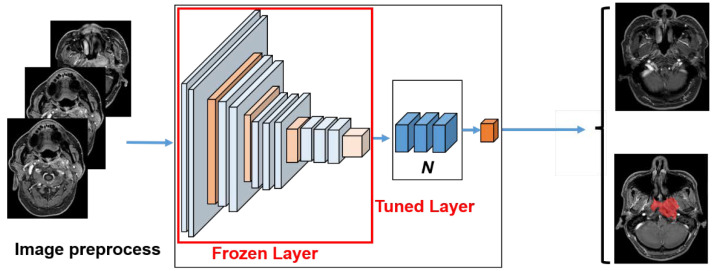
The schematic figure of the model.

Shallow and deep CNN models were selected to identify the optimal classifier for NPC images. The parameters in each layer of ResNet and EfficientNet models were optimized for the ImageNet dataset. Due to the difference in the domains of the ImageNet and our NPC images database, we could not directly use the pre-trained parameters from the trained model on the ImageNet. For our application, one new layer for our case replaced the top layer. For binary classification, we realized the necessity to add one dense layer at the top of each model after removing the top layers from each model. The modified model is shown in Figure 2. We performed the fine-tuning process for the model. We tried to leverage the learned knowledge from the models trained on the ImageNet dataset. In the fine-tuning step, the parameters-trained model was tuned to adapt to the new image dataset of NPC. The fine-tuning was performed in order to obtain some parameters for the last few layers of a pre-trained model while keeping the parameters of the frozen layers. The number of last layers was optimized.

The following CNN-based models are evaluated.

**CNN:** The shallow CNN is hereby referred to as sCNN. The sCNN consists of three 2D convolution layers, three max-pooling layers, and two dense layers. We aimed to identify one deep CNN for the diagnosis of NPC. The sCNN is built by 3 convolutional layers as the baseline model in order to show the improvement from shallow to deep CNN models. We did not build a model with more than 3 convolutional layers considering the visualization for Grad-CAM. The filters on the convolution layers were 32, 64, and 128. The kernel size was set as (3, 3) and the pool size of three max-pooling layers was set as (2, 2). A dropout of 0.5 was used to improve the generalization ability of sCNN. A batch size of 24 was used in the training and validation process. The number of neurons in the dense layer, the activation, and the learning rate was optimized by the Bayesian hyper-parameter optimization method.

**Figure 3:**
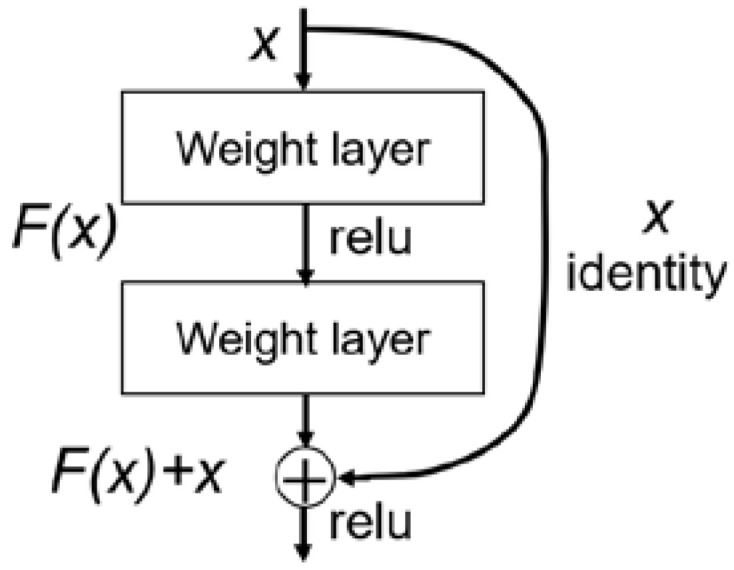
The building block of ResNet.

**ResNet50 and ResNet101**: The representative residual learning architectures ResNet50 and ResNet101 were selected [38]. The output layer was modified for the binary classification using “Sigmoid” activation. The last several layers of ResNet will be trained on our dataset. The number of trainable layers, the activation, and the optimizer were chosen in order to optimize by the Bayesian hyper-parameter optimization method. We also compared the effects of whether or not to include pre-trained weights of “ImageNet” in this study. The advantage of ResNet is that it can train a deeper network and alleviate the problem of gradient disappearance due to its special connection structure. Take one block of ResNet for example (Figure 3), *H(x)* is the desired mapping to fit some stacked layers. *F(x)* is the residual function for the network layer:

(1) F(x)=H(x)-x

Because *H(x)* is more difficult to learn than *F(x)*. We can recast the original mapping to F(x) + x.

(2) H(x)=F(x)+x

As proved by He et al., ResNet can easily train deeper networks than other feedforward networks [38].

**EfficientNet-B7:** EfficientNet is one type of scaled CNN that carefully balances network depth, width, and resolution [39]. EfficientNet achieves better top-1 accuracy on ImageNet and CIFAR-100, etc. EfficientNet models were scaled from baseline EfficientNet-B0 up to EfficientNet-B7 by scaling the coefficients of network width, depth, and resolution. Because EfficientNet-B7 achieves the highest top-1 accuracy in the ImageNet task, we selected EfficientNet-B7 for our evaluation. The number of trainable layers, the activation, and the optimizer were further optimized.

The “ImageNet” weights provided the basic parameters for deep neural networks. Whether pre-trained weights can improve model robustness is still under debate [40,41]. Thus, we also examined whether pre-trained weight is beneficial for our image classification task. Early stopping was used to decide when to stop training by monitoring the loss of the validation set. All models were built by TensorFlow (TensorFlow v2.6.0. https://anaconda.org/conda-forge/tensorflow/files?version=2.6.0 (accessed on 16 November 2021)) and Keras (Keras v2.6.0. https://anaconda.org/conda-forge/keras (accessed on 16 November 2021).

### 2.4. Hyper-parameter Optimization

Bayesian hyper-parameter optimization was used to search the hyper-parameters for shallow and deep CNN. We first optimized the hyper-parameters of sCNN, ResNet50, ResNet101, and EfficientNet-B7 on one randomly selected dataset. Parameter space was assigned following the Gaussian process because the choice can be smartly made to choose the next parameter to evaluate by the acquisition function over the Gaussian prior. The fitness value can be defined according to the requirement. The negative value of validation accuracy of models was used as the acquisition function in order to minimize during each cycle of hyper-parameter optimization. The performance scores were calculated in each loop until the acquisition function did not improve. The Scikit-optimize 0.9.0 package was used to conduct the hyper-parameter optimization process.

### 2.5. Data Analysis

The performance of the model was evaluated using precision, accuracy, F1-score, sensitivity, and specificity. The precision and accuracy are defined as:

(3) $$\mathrm{Precision}=\frac{\mathrm{TP}}{\mathrm{TP}+\mathrm{FP}}$$

(4) $$\mathrm{Accuracy}=\frac{\mathrm{TP}+\mathrm{TN}}{\mathrm{TP}+\mathrm{FP}+\mathrm{TN}+\mathrm{FN}}$$

where TP, TN, FP, and FN are the numbers of true positive, true negative, false positive, and false negative detections, respectively. F1-score is the harmonic average of precision and recall, which is defined as follows:

(5) $$\mathrm{Recall}=\frac{\mathrm{TP}}{\mathrm{TP}+\mathrm{FN}}$$

(6) $$F1\text{-}\mathrm{score}=\frac{2\times \mathrm{TP}}{2\times \mathrm{TP}+\mathrm{FN}+\mathrm{FP}}$$

Sensitivity and specificity are evaluation metrics that show the ability of a model to correctly classify a person as a patient or normal control. Sensitivity refers to the model’s ability to designate an individual with the disease as positive. It is also called the true-positive rate.

(7) $$\mathrm{Sensitivity}=\frac{\mathrm{TP}}{\mathrm{TP}+\mathrm{FN}}$$

Specificity is defined as the ability of the model to correctly identify a person who does not have the disease. It is also called the true negative rate.

(8) $$\mathrm{Specificity}=\frac{\mathrm{TN}}{\mathrm{FP}+\mathrm{TN}}$$

The AUC score indicates how well the model can distinguish between classes. This score is usually adopted to assess a binary classification task. The range of the AUC score is from 0 to 1. A model having an AUC score close to 1 is considered the best model. The AUC score is calculated as follows:

(9) $$AUC=\frac{1}{mn}\sum_{i=1}^{m}\sum_{j=1}^{n}1_{p_{i}>p_{j}}$$

Here *i* runs over all m data points with true label 1, and *j* runs over all n data points with true label 0; $p_{i}$ and $p_{j}$ denote the probability score assigned by the classifier to data point *i* and *j*, respectively.

The confusion matrix was used to visualize the performance of the evaluated models, which comprises four combinations of prediction and ground truth.

The k-fold cross-validation method was used to calculate the evaluation metrics. To reduce statistical bias induced by a chance encounter of single learning, the models were conducted 50 times by using a different initialization of neural networks and different splitting seed. The memory of the trained model was cleared after each cycle.

## 3. Results

### 3.1. Hyper-Parameter Optimization

**Figure 4:**
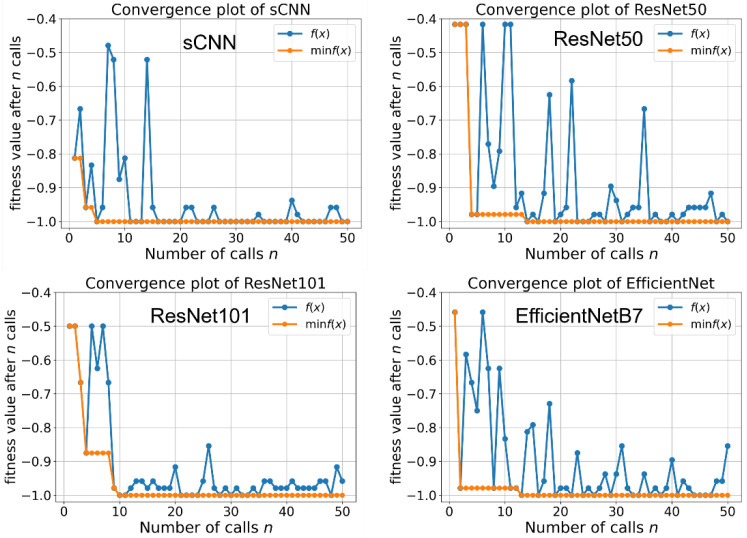
Convergence plot of each model during hyper-parameter optimization.

We trained shallow and deep convolutional neural networks on our collected NPC image dataset. First of all, we tuned the hyper-parameters of each model using the Bayesian optimization process. The hyper-parameter of sCNN, ResNet50, ResNet101, and EfficientNet-B7 were optimized on one train-test split set by using the same random seed for the model_selection method in the Scikit-learn package. We trained the model on Nvidia Geforce RTX 3070 with 8 GB GPU memory. The batch size was set as 24. The optimization processes take 3.6 h and 7.5 h for ResNet and EffficientNet, respectively. For the training process, the accuracy and loss of the validation dataset are used to monitor the course of training. The loss is computed according to binary cross-entropy. The network is re-trained from scratch. As shown in Figure 4, the fitness values suggested that the optimal hyper-parameters can be achieved within 50 runs. The min f(x) is the lowest value that has been obtained in the number of calls in the Bayesian optimization process. In this study, 50 calls were used to get the converged lowest f(x). The hyper-parameter corresponding to the lowest f(x) was chosen for the CNN models. The hyper-parameters of each model are listed in Table S1 in the supporting information.

**Figure 5:**
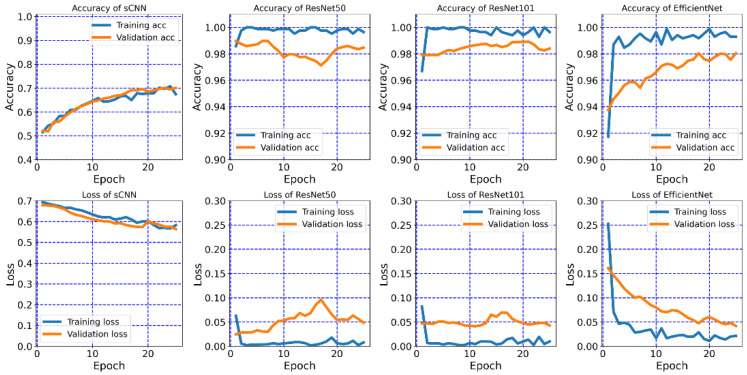
Accuracy and loss of each model on the training and validation dataset.

We validated the performance of each model by monitoring the accuracy and loss. Figure 5 shows the accuracy and loss of models on the training and validation datasets in the 25 epochs of training. The training process was stopped when the validation accuracy did not improve further by using the early stopping method. The small deviation between the accuracy of the training set and the accuracy of the validation set was in the reasonable region, suggesting that the models were not over-fitted in the 25 epochs. From the statistical average of 50 rounds of k-fold calculation, the validation accuracy values of three deep neural networks including ResNet50, ResNet101, and EfficientNet-B7 reached 0.98, outperforming the sCNN with a validation accuracy of 0.73. The loss of three deep neural networks was as low as 0.05 while it was 0.57 for sCNN. Moreover, Deep neural networks including ResNet50, ResNet101, and EfficientNet-B7 quickly reached the converged results within 10 epochs and sCNN required 22 epochs, suggesting that the three deep neural networks are effective in learning. In the training process, we found that three hierarchical CNN models achieved a higher accuracy in both training and validation datasets than the sCNN. Among the examined models, ResNet50 gave the smoothest training accuracy, indicating the quick learning ability of ResNet50 on the NPC image dataset. For a fair comparison, we used 25 epochs for all of the models in the following training.

### 3.2. Performance of Shallow and Hierarchical Learning

**Figure 6:**
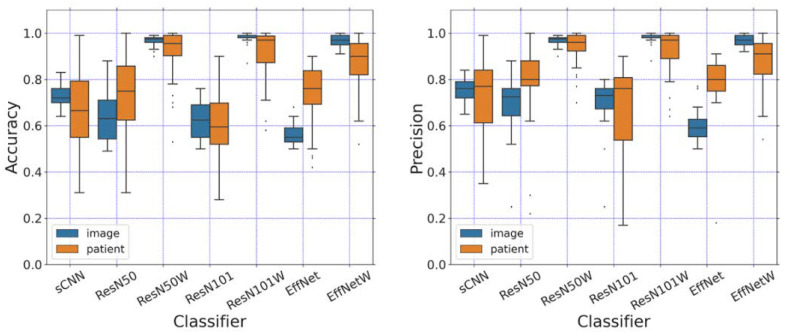
Precision and accuracy of each model on the test dataset. The models without pre-trained weights are labeled as ResN50, ResN101, and EffNet for ResNet50, ResNet101, and EfficientNet-B7. The models using pre-trained weights are labeled as ResN50W, ResN101W, and EffNetW for ResNet50, ResNet101, and EfficientNet-B7.

To further validate the performance, we computed the evaluation metrics of shallow and deep neural networks. We examined the effects of two factors on the performance: the choice of splitters and the transfer learning of the pre-trained weights. The performance was validated by varying the initialization of models and the random seed of the train/test splitter in 50 individual runs. The accuracy, precision, and F1-score are shown in Figure 6 and the statistical average is summarized in Table 1 and Table 2.

### 3.3. Choice of Splitter Affects Performance

The image- and patient-level splitters were examined by using the same set of hyper-parameters. Though the section gap was adopted between neighboring slices in the MRI image series, we could not overlook that consecutive images stored in the MRI series shared a higher similarity. The image-level splitter was expected to yield better performance when neighboring slices were divided into train and test datasets.

**Figure 7:**
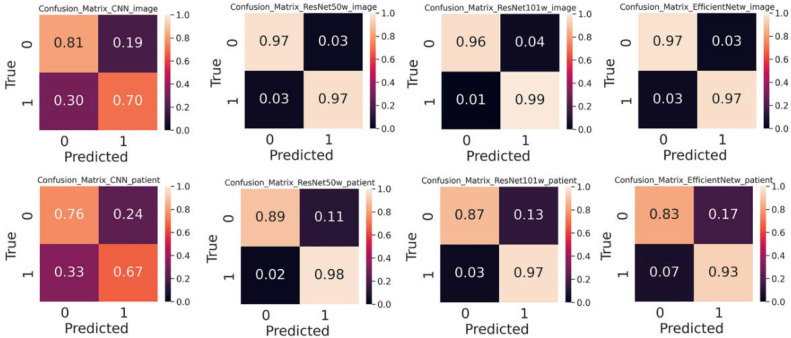
Confusion matrix of each model on the test dataset using image and patient-level splitter. The pre-trained weights are included. In each sub-figure, 0 is labeled for the patient and 1 is labeled for the normal.

Figure 6 and Table 1 show that the image-level splitter reproduces both higher accuracy and precision than the patient-level splitter. The confusion matrix shown in Figure 7 represents the classification performance in differentiating non-NPC and NPC images. The confusion matrix shows that the classification probability for the image-level splitter is higher than that of the patient-level splitter for all four CNN models. The choice of splitter gives less influence on ResNet50, indicating the robustness of the model.

The image-level diagnosis determines the incurrence of NPC based on a single image. To remove the bias introduced by neighboring images, the patient-level splitter was used to divide the train/test dataset. The patient-level splitter should then provide a reliable prediction, which will reveal the true prediction performance of the examined models. As shown in Table 2, ResNet50 with pre-trained weights (referred to as ResNet50-Weight), respectively, achieves accuracy, precision, and an F1-score of 0.97, 0.97, 0.97, which would be the optimal model for the classification. ResNet101-Weight gives a slightly higher average value but wider distribution as shown in Figure 6, meaning that the reliability is slightly lower than ResNet50-Weight. As revealed from the evaluation metrics, ResNet50-Weight provides the highest value of accuracy, precision, F1-score, sensitivity, and specificity. ResNet50-Weight can classify both the true positive and true negative images. From the average value and the distribution, we can see that the ResNet50-Weight is a suitable DL-based model for NPC image classification.

### 3.4. Pre-trained Weight Improves Performance

Pre-training weights trained on the large image dataset “ImageNet” can be transferred to the image classification. Without pre-trained weights, ResNet50, ResNet101, and EfficientNet-B7 displayed a slightly higher classification performance than sCNN as shown in Figure 7 and Figure S1. After including pre-trained weights, all three deep learning models reached a higher precision and accuracy than sCNN, leading to the best accuracy of 0.93 achieved by the ResNet50-Weight. As shown in Figure 7, ResNet50 is capable of differentiating non-NPC and NPC images with accuracies of 0.89 and 0.98 by using the patient-level splitter. The pre-trained weights were beneficial for our medical image classification though the weights were pre-trained on the general image classification task of “ImageNet”.

To make a comparison with the previous machine learning-based model, we computed the area under the curve (AUC), which was summarized in Table 1. The AUCs of ResNet50-Weight, ResNet101-Weight, and EfficientNet-B7-Weight were 0.97, 0.98, 0.97, respectively. The AUCs were higher than 0.846 when obtained by RF using the selected features of MRI [23]. Note that the value between our model and previous reports cannot be compared directly because the computational details and datasets are not exactly set the same. However, the result indicates that our model achieves comparable performance as shown in Table 3. Further validation of our proposed model can be examined with other public datasets. The result implies that deep CNN can get comparable performance over traditional machine learning methods in the classification of NPC images without feature selection.

It is worth noting that a deeper neural network does not always guarantee higher accuracy and precision in our study. The fine-tuned ResNet50-Weight reached the best accuracy of 0.93, which is higher than that of the ResNet101-Weight (0.91) and the EfficientNet-B7-Weight (0.87). Though EfficientNet-B7 achieved the highest top-1 accuracy in the task of “ImageNet”, the optimal prediction model was the ResNet50 in the NPC classification task. Therefore, deep learning models should be self-tested on a case-by-case basis. The optimal model needs to be identified based on the specified tasks.

### 3.5. Interpretability of Shallow and Hierarchical Learning

To obtain the interpretability of the diagnosis of DL methods, a gradient-weighted class activation map (Grad-CAM) was used to visualize the feature map [45]. Grad-CAM was used to understand which regions of the image affect the prediction by projecting back the weights of the convolutional layer back to the input images. The convolutional layers adjacent to the output layers of sCNN, ResNet, and EfficientNet-B7 were generated for feature representation. 

**Figure 8:**
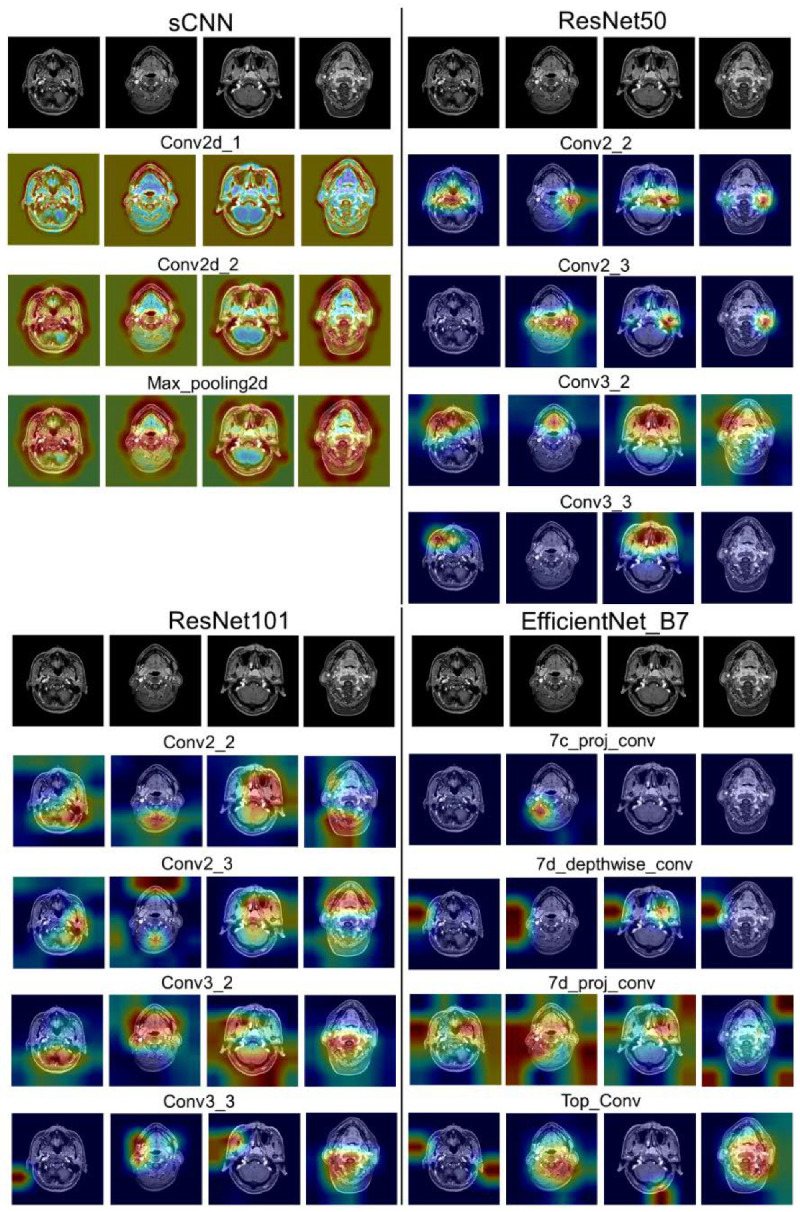
Feature maps for sCNN, ResNet50, ResNet101, and EfficientNet-B7 for normal control and patient. Left two for normal and right two for patients. The top row is for the original images and the bottom row is for the convolutional layers adjacent to the output layers.

The feature maps in Figure 8 visualize the regions that most influence the model’s decision in the selected testing images by superimposing the heatmap on the original images. The whole nasopharynx space includes the nasopharynx, the fibrofatty parapharyngeal space, the pterygoid bodies, and the anterolateral plates. NPC causes thickening and asymmetry in the nasopharynx, thus more weights should be concentrated around the nasopharynx space. Two images with an asymmetry in the nasopharynx were selected for visualization. From the feature maps of Figure 8, we can notice that sCNN can contour the boundary between the skull and background, whereas ResNet50-Weight, ResNet101-Weight, and EfficientNet-B7-Weight provide more weights on localized regions. ResNet50 gives more concentrated weights on the site of the parapharyngeal space, which is the common occurrence site of NPC. Conv2_2 and conv2_3 of ResNet50-Weight put more weight on the lateral nasopharyngeal walls and eustachian tubes. Among the three deep CNN models, the weights of ResNet50 are more concentrated, explaining why ResNet50-Weight outperforms over two deeper models, ResNet101-Weight and EfficientNet-B7-Weight.

## 4. Discussion

Increasing interest has been directed to AI-assisted medical diagnosis. However, the latest deep CNN has not yet been adopted in the AI-assisted diagnosis of NPC. The classification performances of deep and shallow CNN have not been quantitatively compared. In this study, we constructed a small but high-quality NPC image dataset and evaluated four popular CNN-based AI tools in the classification of NPC images of control and patients. Though the sample size of our dataset was small, it can provide valuable information. We demonstrated that hierarchical neural networks including ResNet50, ResNet101, and EfficientNet-B7 can achieve a better performance than that of sCNN in the classifying of non-NPC and NPC based on 50 rounds of k-fold calculation. Moreover, the results indicate that the pre-trained weights can be transferred to our NPC classification task. To interpret the different performances of each DL model, we generated feature maps for convolutional layers adjacent to the output layers. The ResNet50-Weight put more weight on the localized region that is closer to the areas of the common occurrence site of NPC. In summary, the effectiveness in learning comes from the deep architecture of the model, and the higher accuracy benefited from the pre-trained weights for our classification task.

Two limitations of our study are worthy of improvement. First, the dataset was not large enough in comparison to other medical image datasets, such as the chest X-ray dataset. As the preliminary dataset of NPC images, the models were trained to classify non-NPC and NPC images without conducting staging. To advance the effectiveness and reliability of deep CNN-based models, more patients from additional trials will be continuously collected. Second, our proposed DL models utilized MRI imaging information. In the future, clinical and biopsy information (e.g., stage, segmentation, and lesion images) can be incorporated into other DL models in order to further improve the robustness and prediction performance.

## 5. Conclusions

We examined the classification performance of deep hierarchical and simple shallow CNN models on our tailored NPC image dataset. By fine-tuning the networks of ResNet50, ResNet101, and EfficientNet-B7, we obtained higher accuracy than shallow CNN. Particularly, ResNet50 with pre-trained weight achieved the highest precision, accuracy, F1-score, sensitivity, and specificity, displaying the best classification performance. From visualization using Grad-CAM, ResNet50 gave more concentrated weights on the site of the parapharyngeal space in images, which is the common occurrence site of NPC. Therefore, ResNet50 was identified as the optimal deep CNN-based model for the potential support of clinical decisions in the diagnosis of NPC. Hopefully, the DL models will be integrated into clinical practice to provide supplementary and quantitative information on the early diagnosis of NPC tumors.

## Figures and Tables

**Table 1:** Evaluation metrics of each model by using an image-level splitter.

	Accuracy	Precision	F1-Score	AUC
**sCNN**	0.72	0.75	0.72	0.73
**ResNet50**	0.64	0.67	0.59	0.64
**ResNet50-Weight**	0.97	0.97	0.97	0.97
**ResNet101**	0.62	0.68	0.57	0.63
**ResNet101-Weight**	0.98	0.98	0.98	0.98
**EfficientNet**	0.56	0.60	0.52	0.56
**EfficientNet-Weight**	0.97	0.97	0.97	0.97

**Table 2:** Evaluation metrics of each model by using the patient-level splitter.

	Accuracy	Precision	F1-Score	AUC	Sensitivity	Specificity
**sCNN**	0.67	0.72	0.64	0.66	0.78	0.54
**ResNet50**	0.74	0.82	0.71	0.76	0.66	0.86
**ResNet50-Weight**	0.93	0.94	0.93	0.94	0.90	0.98
**ResNet101**	0.61	0.66	0.55	0.63	0.76	0.97
**ResNet101-Weight**	0.91	0.93	0.91	0.98	0.87	0.97
**EfficientNet**	0.75	0.79	0.73	0.76	0.72	0.80
**EfficientNet-Weight**	0.87	0.88	0.87	0.87	0.83	0.91

**Table 3:** Accuracy of recently reported models.

Model	Image	Accuracy
**Inception** [42]	endoscopy	0.89
**ANN** [29]	microscopy	0.93
**ANN** [28]	endoscopy	0.92
**CNN** [43]	MRI	0.91
**CNN** [34]	endoscopy	0.95
**Residual Attention** [44]	MRI	0.92
**ResNet50-Weight**	MRI	0.93

## Data Availability

The datasets generated during the current study are stored in the Open Science Framework https://osf.io/42x7d/?view_only=6b78b450cb3f409dae9226eff4a6fcb4 (accessed on 1 April 2022). The scripts used in this study can be found in the online repositories https://github.com/xlxgit/NPC_Classify (accessed on 1 April 2022).

## Supplementary Materials

The following supporting information can be downloaded at: https://www.mdpi.com/article/10.3390/diagnostics12102478/s1, Table S1: Optimized hyper-parameters; Figure S1: Confusion matrix of each model on the test dataset using the image and patient-level splitter.
