# Supplementary material for: Deep Convolutional Neural Network for Nasopharyngeal Carcinoma Discrimination on MRI by Comparison of Hierarchical and Simple Layered Convolutional Neural Networks
Source: Diagnostics (Basel). 2022 Oct 13;12(10):2478. doi: 10.3390/diagnostics12102478 (PMC9601165; doi:10.3390/diagnostics12102478)
Supplement: Supplementary file 1 [file diagnostics-12-02478-s001.zip › diagnostics-1931248-supplementary.pdf]

Deep Convolutional Neural Network for Nasopharyngeal Carcinoma Discrimination on MRI by Comparison of Hierarchical and Simple Layered Convolutional Neural Networks

Li Ji [1], Rongzhi Mao [2], Jian Wu [1], Cheng Ge [2], Feng Xiao [1], Xiaojun Xu [2,*] , Liangxu Xie [2,*] and Xiaofeng Gu [1,*]

[1] Department of Otorhinolaryngology, The Second People's Hospital of Changzhou affiliated to Nanjing Medical University, Changzhou 213003, China
[2] Institute of Bioinformatics and Medical Engineering, School of Electrical and Information Engineering, Jiangsu University of Technology, Changzhou 213001, China
* Correspondence: xiaofenggu2006@163.com (X.G.); xuxiaojun@jsut.edu.cn (X.X.); xieliangxu@jsut.edu.cn (L.X.)

TABLE S1. Optimized hyper-parameters.

Model	Hyper-parameters
**CNN**	Learning_rate=0.000205159; num_dense_nodes=16; activation=sigmoid
**ResNet50**	Num_trainable_layer=15；optimizer=adam, activation = sigmoid
**ResNet50_weight**	Num_trainable_layer=10；optimizer=adam, activation = sigmoid
**ResNet101**	Num_trainable_layer=20 ； optimizer=sgd, activation = sigmoid
**ResNet101_weight**	Num_trainable_layer=10；optimizer=adam, activation = sigmoid
**EfficientNetB7**	Num_trainable_layer=12；optimizer=adam, activation = sigmoid
**EfficientNetB7_weight**	Num_Trainable_layer=4；optimizer=adam, activation = sigmoid

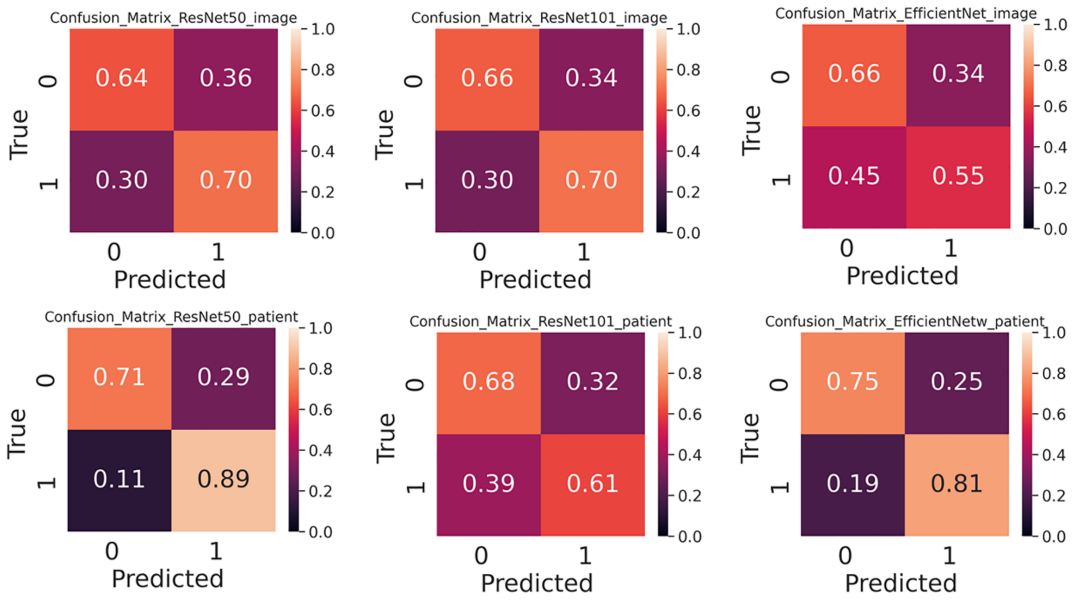

FIGURE S1 Confusion matrix of each model on the test dataset using image and patient level splitter. The pre-trained weights are not included.
